# Cellular Immunological Memory T Cells and *IL15RA* Gene Polymorphism in COVID-19 Vaccinated Individuals from Southern Brazil

**DOI:** 10.3390/diagnostics16010089

**Published:** 2025-12-26

**Authors:** Grazielle Motta Rodrigues, Pâmela Portela da Silva, Maria Clara de Freitas Pinho, Taís da Silveira Fischer, Fernanda de Paris, Fabrício Souza Campos, Arthur Bandeira de Mello Garcia, Lucas Fernandes Jataí, Patricia Ashton-Prolla, Fernanda Sales Luiz Vianna, Clévia Rosset

**Affiliations:** 1 Postgraduate Program in Medical Sciences, Federal University of Rio Grande do Sul, Porto Alegre 90010-150, Brazil; gmorodrigues@hcpa.edu.br; 2 Genomic Medicine Laboratory, Porto Alegre Clinical Hospital, Porto Alegre 90035-903, Brazil; mcfpinho_est@hcpa.edu.br (M.C.d.F.P.); tsfischer@hcpa.edu.br (T.d.S.F.); abmgarcia@hcpa.edu.br (A.B.d.M.G.); lucafjatai@gmail.com (L.F.J.); 3 Laboratory Diagnostic Service, Porto Alegre Clinical Hospital, Porto Alegre 90035-903, Brazil; pporsilva@hcpa.edu.br (P.P.d.S.); fparis@hcpa.edu.br (F.d.P.); 4 Department of Microbiology, Immunology and Parasitology, Federal University of Rio Grande do Sul, Porto Alegre 90010-150, Brazil; camposvet@gmail.com; 5 Medical Genetics Service, Porto Alegre Clinical Hospital, Porto Alegre 90035-903, Brazil; pprolla@hcpa.edu.br

**Keywords:** cellular memory, *IL15RA*, SARS-CoV-2, vaccines, T cell

## Abstract

**Background:** The development of safe and effective vaccines against SARS-CoV-2 was crucial for controlling COVID-19 and establishing long-lasting immune memory in the population. **Methods:** This study evaluated cellular immune memory in individuals vaccinated with different regimens in Rio Grande do Sul using flow cytometry. Additionally, the rs2228059 polymorphism in the *IL15RA* gene was genotyped. A total of 62 participants were randomly recruited. **Results:** A decrease in memory T cell subsets in response to SARS-CoV-2 stimuli was observed in total CD3^+^, CD4^+^, and CD8^+^ T cells. Regarding the timing of the last vaccine dose, 94.4% of participants had received their final COVID-19 vaccination at least two years prior to recruitment. The rs2228059 polymorphism was genotyped in 443 individuals from the Rio Grande do Sul population. Among participants who received the ChAdOx1/ChAdOx1/BNT162b2 vaccination regimen and carried the TT genotype, an increase in CD8^+^ naive, CD8^+^ effector and CD4^+^ naive subsets was observed in stimulated cells. Although preliminary, the results suggest no major differences between vaccination regimens. **Conclusions:** The progressive reduction in memory T cell counts supports the need for booster doses, which is essential not only in the context of new emerging variants but also especially to maintain adequate cellular immune protection.

## 1. Introduction

SARS-CoV-2, first identified in Wuhan at the end of 2019, is the virus that causes COVID-19, a respiratory disease with symptoms ranging from mild to severe [1]. Globally, the rapid spread of COVID-19 has resulted in over 700 million cases, posing a significant public health challenge [2]. In Brazil, by January 2025, over 39 million cases and over 700 thousand deaths from COVID-19 have been reported, and multiple vaccines using mRNA, inactivated virus, and adenovirus vector technologies have been introduced [3]. The development of effective and safe vaccines has profoundly impacted the course of the pandemic, becoming a critical strategy for preventing and controlling disease transmission by promoting immune memory formation within the population [3,4].

Brazil was among the countries most severely affected by the COVID-19 pandemic, with widespread population exposure to SARS-CoV-2. Despite the public health crisis, Brazil was one of the leading nations in vaccine administration, delivering a significant number of doses by June 2021, and over 550 million doses by January 2025 had been administered through the Brazilian public health system [3,5]. The two primary vaccines used to immunize the Brazilian population were CoronaVac (Sinovac COVID-19 vaccine) and ChAdOx1-S (AstraZeneca-Oxford COVID-19 vaccine) [6]. Additionally, other COVID-19 vaccines, including Ad26.COV2.S (Janssen) and BNT162b2 (Pfizer-BioNTech), were approved by national health surveillance agencies for primary immunization. This approval enabled various heterologous booster vaccination combinations, further enhancing immunization efforts [7].

SARS-CoV-2 infection or vaccination triggers a broad activation of both the innate and adaptive immune systems in humans. The adaptive immune response involves both humoral and cellular components, enabling long-term protection against severe COVID-19 through the formation of immunological memory [8]. Typically, the presence of antibodies in serum samples is used as a surrogate marker for predicting protection against a pathogen. Studies have assessed humoral protection against SARS-CoV-2 evaluating neutralizing antibody levels [9,10]. However, the role of cellular immunity has gained increasing attention. A few studies have analyzed clinical cohorts in the context of pre- and post-SARS-CoV-2 infection, revealing a reduction in CD4^+^ and CD8^+^ T-cell counts after infection, which may affect the formation and maintenance of immunological memory [11,12]. One study investigated differences in immune memory formation induced by three vaccine platforms (mRNA, viral vector-based, and protein-based adjuvanted vaccines) compared to natural infection. The data demonstrated that, although antibody levels decline over time following vaccination, memory T and B cells remain relatively stable, supporting long-lasting immune protection [13]. Another study found that hybrid immunity (natural infection combined with vaccination) provides durable protection, maintaining immune responses even three years post-infection or vaccination. This includes the development of T cells specific to the spike protein and other viral antigens [14]. Thus, the presence of immunological memory, particularly cellular memory, is critical for achieving effective population-level immunity. Research on the longevity of cellular immune responses could offer useful information for optimizing and monitoring vaccination strategies, especially in middle-income countries like Brazil, where healthcare resources are limited. To date, no studies have evaluated differences in long-term cellular protection conferred by heterologous booster vaccines in Brazil.

In addition to variable exposure to the virus and individual lifestyle factors, genetic variants may also influence the formation and persistence of immunological memory. The single nucleotide polymorphism (SNP) rs2228059 is located in the *IL15RA* gene and results in a nonsynonymous amino acid substitution from asparagine to threonine at position 182 of the protein (p.Asn182Thr, N182T) [15]. According to variant annotation databases, this substitution is classified as likely benign/benign (LB/B), suggesting no major deleterious impact on protein function [16]. The rs2228059 variant is relatively common worldwide, with a minor allele frequency (MAF) of approximately 0.48 according to the 1000 Genomes Project (Phase 3), remaining similar among European, American, East Asian, and South Asian populations, with lower frequency observed in African populations (0.24) [17]. This information indicates that this polymorphism represents a frequent genetic variant rather than a rare mutation. The rs2228059 T>G polymorphism has been associated with differences in the binding affinity of IL-15, a key cytokine that regulates the basal proliferation of memory immune cells [18]. Some studies suggest that this variant may exert modest modulatory effects in specific immunological contexts, including a potential increased risk of kidney transplant rejection [19,20]. The Brazilian population has a highly diverse genetic background [21], but the frequency of this polymorphism has not yet been described in genomic datasets for this population [22].

This study employs two complementary approaches to evaluate immunological memory in a population from southern Brazil. The first approach involves describing and quantifying SARS-CoV-2-specific T-cell immunological memory in individuals who underwent different vaccination regimens in Rio Grande do Sul. The second approach assesses the frequency of the rs2228059 polymorphism both in these individuals and in an independent cohort. Therefore, the objective of this study was to investigate the status of immunological memory and to explore potential genetic factors influencing its persistence in this population.

## 2. Materials and Methods

### 2.1. Study Participants and Ethical Considerations

A total of 62 adult participants (over 18 years old) were recruited between August/2023 and September/2024 from the Hospital de Clínicas de Porto Alegre (HCPA) in Porto Alegre, Rio Grande do Sul, Brazil. Participants were categorized into three groups according to their initial SARS-CoV-2 vaccination regimens. These regimens were applied as inclusion criteria: Group 1 (*n* = 23): First and second doses of CoronaVac (Sinovac COVID-19 vaccine) followed by a booster dose of BNT162b2 (Pfizer-BioNTech COVID-19 vaccine). Group 2 (*n* = 24): First and second doses of ChAdOx1-S (AstraZeneca-Oxford COVID-19 vaccine) followed by a booster dose of BNT162b2 (Pfizer-BioNTech COVID-19 vaccine). Group 3 (*n* = 7): First three doses of BNT162b2 (Pfizer-BioNTech COVID-19 vaccine). The study was approved by the local ethics board (CAAE number: 60924322.0.0000.5327), and all participants provided written informed consent covering both the cellular analyses and the genotyping procedures. A questionnaire was administered to gather self-reported information on COVID-19 symptoms, prior SARS-CoV-2 infections, previous testing (PCR or antigen), and other vaccinations received close to the time of recruitment.

### 2.2. PBMC Isolation and Culture

Peripheral blood samples were collected from each SARS-CoV-2 vaccinated individual at a single time point during recruitment using one EDTA-containing tube and one serum-separating tube. Peripheral blood mononuclear cells (PBMC) were isolated in the EDTA-containing tube using Ficoll-Paque PLUS density gradient centrifugation (Cytiva/Global Life Sciences Solutions, Marlborough, MA, USA). Four milliliters of whole blood were diluted with an equal volume of 1× PBS and layered onto 4 mL of Ficoll-Paque PLUS in a 15 mL conical tube. The tube was centrifuged at 454× *g* for 30 min with the brake off and a low acceleration rate. After centrifugation, the PBMC-containing interface was collected and transferred to a new 15 mL conical tube containing 4 mL of 1×PBS, followed by centrifugation at 200× *g* for 10 min. The resulting cell pellet was resuspended in 1 mL of RPMI-1640 medium (Gibco, Thermo Fisher Scientific, Waltham, MA, USA, #cat R8758). Cell counts were performed using a Neubauer chamber.

To separate autologous serum from each participant, the serum-separating tube was centrifuged at 10,000× *g* for 5 min. Subsequently, three simultaneous cultures were prepared for each participant: a stimulated culture, an unstimulated culture, and a control. Isolated PBMCs were distributed into three microtubes containing specific reagents: (1) Stimulated Culture: 1 μg/mL of PepTivator SARS-CoV-2 Select premium grade (Milteny Biotec, Bergish Gladbach, Germany, #cat 130-127-309) [23], 1% Penicillin-Streptomycin (Gibco, Thermo Fisher Scientific, Waltham, MA, US, $cat), 20% autologous serum, 1 × 10^6^ isolated PBMCs, and RPMI-1640 medium to a final volume of 200 μL. (2) Unstimulated Culture: 1% Penicillin-Streptomycin, 20% autologous serum, 1 × 10^6^ isolated PBMCs, and RPMI-1640 medium to a final volume of 200 μL. (3) Control Culture: 5 μg/mL of Phytohemagglutinin, M form (PHA-M, Gibco, Thermo Fisher Scientific, Waltham, MA, US, #cat: 10576015), 1% Penicillin-Streptomycin, 20% autologous serum, 1 × 10^6^ isolated PBMCs, and RPMI-1640 medium to a final volume of 200 µL. PHA-M was included in the control culture to evaluate cell expansion in response to a general stimulus. The prepared tubes from each participant were seeded in 96-well culture plates and incubated for 20–24 h at 37 °C with 5% CO_2_ in a humidified incubator. After incubation, the PHA-M wells were examined under a microscope to evaluate cell proliferation by assessing the formation of cell clumps. Low response to the PHA-M was defined as the absence of clump formation. Samples with a low response stimulus would be excluded from subsequent analyses.

### 2.3. Flow Cytometry Assay

**Figure 1:**
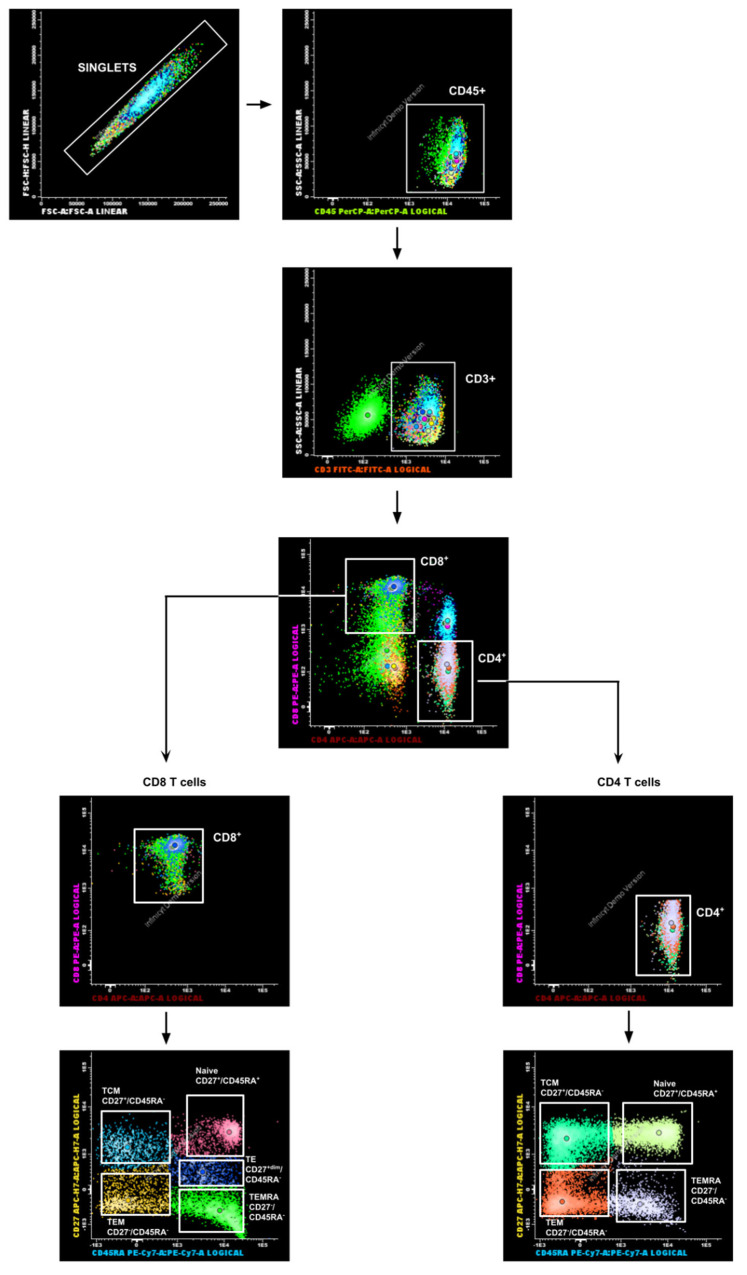
Flow cytometry gating strategy. Representative dot plots showing SARS-CoV-2-specific CD8^+^ and CD4^+^ T cells within the lymphocyte gate. TCM = central memory T cells; TEM = effector memory T cells; TEMRA = effector memory T cells expressing RA^+^; TE = dim effector memory T cells.

After the incubation period, supernatant cells from each well were collected into separate tubes. To retrieve any remaining adherent cells, the plate surface was treated with a 1:1 mixture of 0.25% trypsin containing 0.9 mM EDTA (Invitrogen, Carlsbad, CA, USA, #cat 25200056) and autologous serum, followed by incubation at 37 °C for 2 min. Cells from each well were stained with surface markers using two antibody panels: Panel 1: Anti-CD45, anti-CD3, anti-CD4, anti-CD8, anti-CD45RA, and anti-CD27 to analyze memory T cells. Panel 2: Anti-CD45, anti-CD3, anti-CD4, anti-CD8, and anti-CD69 to assess memory T cell activation. An unstained control tube was also prepared for each sample. Detailed information on staining reagents, catalog numbers, and volumes is provided in Supplementary Table S1. CD45RA and CD27 were used to define T-cell subsets, following the EuroFlow consortium recommendations for standardized immunophenotyping. This approach ensured methodological consistency and alignment with clinically validated flow cytometry protocols. Although other more classical markers such as CD44 and CD62L are also widely used, the combination of CD45RA and CD27 provides equivalent discrimination of T-cell subsets while maintaining compatibility with standardized diagnostic panels. Surface marker staining protocol was also adapted from the EuroFlow protocol [24], for application in cultured cells. For this adaptation, cultured cells were used in place of peripheral blood cells. After the incubation period, cells were harvested using a trypsinization protocol, collected into microtubes, centrifuged, and the supernatant was discarded. The resulting cell pellet was resuspended in PBS, and surface cell staining was performed following the protocol. Further details are provided in Supplementary Material Section S2. Samples were acquired using a BD FACSMelody cell cytometer (BD Biosciences, Franklin Lakes, NJ, USA) with BD FACSChorus software v5.0. For each sample, the unstained control tube was acquired first. A minimum of 10,000 events within the lymphocyte gate was recorded for each tube. The gating strategy for lymphocyte acquisition is shown in Figure 1. FCS files were exported and analyzed using Infinicyt™ software v2.0 (Cytognos S.L., Santa Marta de Tormes, Spain).

### 2.4. rs2228059 Polymorphism Genotyping

The genotyping of the rs2228059 polymorphism was performed on all participants included in this study. DNA was extracted from the remaining PBMCs using the PureLink Genomic DNA Mini Kit (Invitrogen Solutions, Waltham, MA, USA, #cat: K182001), following the manufacturer’s instructions. DNA concentration and purity were assessed using a NanoDrop 2000 spectrophotometer (Thermo Fisher Scientific, Waltham, MA, USA). Samples with an A260/280 ratio of ~1.7 and an A260/230 ratio between 2 and 3 were considered acceptable. DNA concentrations were then normalized to 10–15 ng/µL for downstream applications. To estimate the frequency of this polymorphism in the general population, genotyping was also performed on an independent cohort from the HCPA Biobank [25], from the same region where our study cohort was recruited (Porto Alegre, Rio Grande do sul, Brazil). This cohort comprised 381 individuals that has been described as predominantly of European ancestry, with African and Native American contributions, according to the DNA do Brasil study [26]. Detailed information about the HCPABiobank and its collections can be accessed at: https://sites.google.com/hcpa.edu.br/area-do-pesquisador/serviços/cpe/biobanco(accessed on 16 January 2025).

DNA samples from the Biobank cohort were stored at the HCPA Biobank and accessed by our research group for this study. Genotyping was conducted in 96-well PCR optical plates (MicroAmp, Thermo Fisher Scientific, Waltham, MA, US, #cat N8010560) using the TaqMan Genotyping Master Mix and TaqMan SNP Genotyping Assay C1882528_10 (Applied Biosystems, Waltham, MA, USA). Each reaction contained 6 µL of 1× Genotyping Master Mix, 0.6 µL of 20× TaqMan SNP assay probe, nuclease-free water, and 2 µL of DNA (diluted to 10–15 ng/µL), making a final volume of 10 µL. The plates were sealed with ABsolute qPCR Plate Seals (Thermo Fisher Scientific, Waltham, MA, US, #cat AB1170) and processed on a QuantStudio 3 real-time PCR system using standard PCR cycling parameters. Data analysis was performed using Genotyping & Sequence Analysis Software, v4.1, within the Connect Data Analysis Apps (Thermo Fisher Scientific, Waltham, MA, US). Genetic findings were reported following the STrengthening the REporting of Genetic Association Studies (STREGA) guideline [27]. To validate the genotyping results, 33 samples were tested in duplicates, achieving 100% reproducibility. Additionally, two authors independently reviewed the genotyping results and data entry to ensure accuracy.

### 2.5. Set of Controls

Different control strategies were implemented at each experimental stage to ensure methodological consistency. In the cell culture assays, PHA-M was included in wells containing patient cells. The formation of cell clumps following PHA-M stimulation was used as an internal control to verify the nutritional adequacy of the culture medium, as clump formation reflects appropriate conditions for lymphocyte proliferation. For flow cytometry, an unstained tube from each participant was analyzed prior to acquisition of the stained samples, serving both for cytometer calibration and as a negative fluorescence control for accurate gating and comparison. Finally, in the genotyping assays, a subset of 33 samples was randomly selected and reanalyzed to validate homozygous and heterozygous genotype calls for the rs2228059 polymorphism, confirming the reproducibility and accuracy of the genotyping results.

### 2.6. Statistical Analyses

Statistical analyses were performed in IBM SPSS Statistics (version 18 for Windows) and R Statistical Software (v4.1.2; R Core Team 2021). Paired data was analyzed with the Mixed Linear Model and linear regression, to compare differences in quantitative variables. ANCOVA test was employed to assess quantitative outcomes and two-way ANOVA was conducted to evaluate qualitative data. Post hoc power analyses were conducted using effect size estimates (Cohen’s f) and based on observed effect sizes and sample size (α = 0.05). Hardy–Weinberg equilibrium (HWE) was assessed using the chi-square test. Statistical significance was set at *p* < 0.05.

## 3. Results

### 3.1. Descriptive Overview of the Studied Population

Of the 62 participants randomly recruited for this study, eight were excluded from the flow cytometer analysis due to an insufficient number of lymphocytes acquired (<14,000). The final analysis included 54 individuals, all of whom resided in and received their vaccine doses in Rio Grande do Sul, the southernmost state of Brazil. The majority of participants reported a prior COVID-19 infection, and none exhibited COVID-like symptoms at the time of recruitment (Table 1). The study population comprised 13 males and 41 females, with ages ranging from 18 to 73 years (Table 1). Age and sex were evenly distributed among the vaccination groups (*p* = 0.7897 and *p* = 0.500, respectively). The median time (±standard deviation) since the last vaccine dose was 9 (±7), 13 (±5), and 14 (±6) months for groups 1, 2, and 3, respectively.

### 3.2. T Cell Subsets in the Vaccination Groups

**Figure 2:**
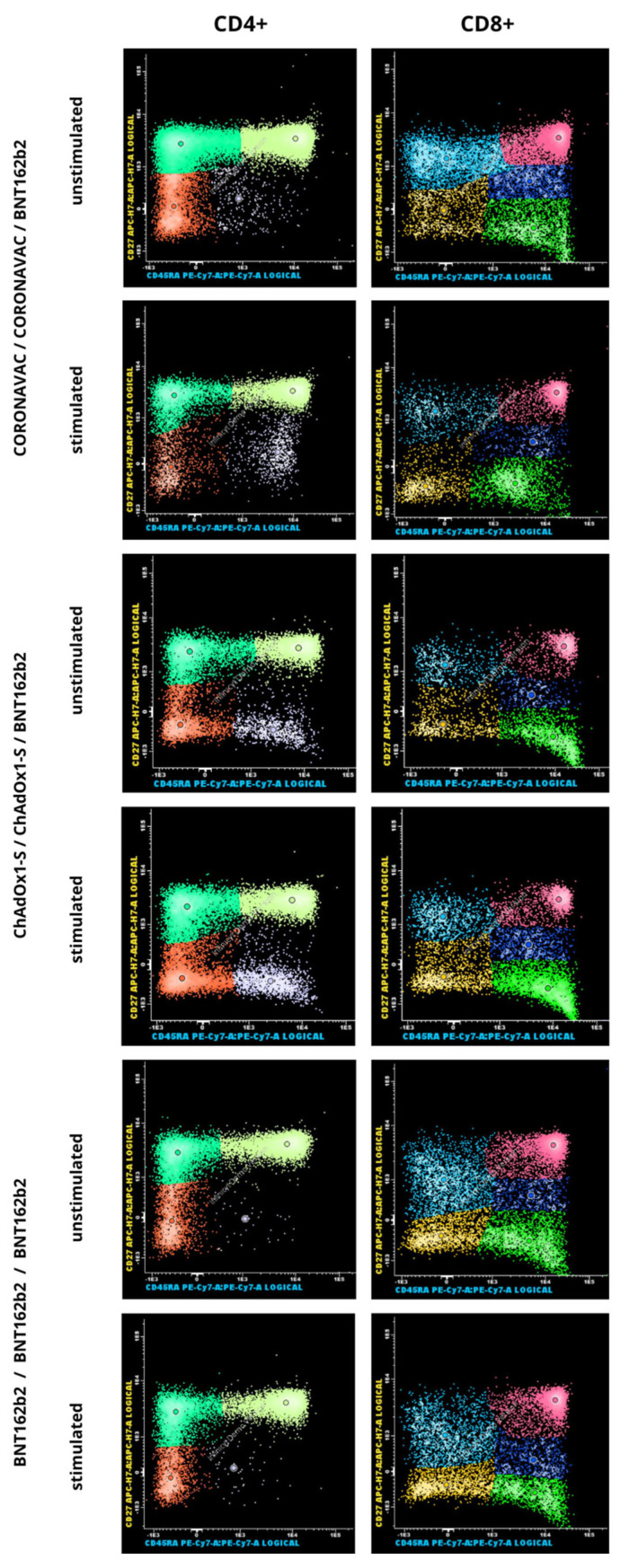
Representative flow cytometry gating strategy for CD4^+^ and CD8^+^ T cells from stimulated and unstimulated cultures, based on surface expression markers CD27 and CD45RA. The gating illustrates the identification and differentiation of T cell subsets, including naive, TCM, TEM, and TEMRA cells, under both culture conditions.

When analyzing the differences in the mean percentage of T cell subsets between stimulated and unstimulated cell cultures from all participants, a reduction in T cell subsets was observed in the presence of stimulus (Table 2 and Table 3). This reduction was statistically significant for mean total T lymphocytes CD3^+^ (*p* = 0.005), CD4^+^ (*p* = 0.007), CD8^+^ (*p* = 0.016), and the subsets CD4^+^ naive (*p* = 0.045), CD4^+^ TCM (*p* = 0.021), CD4^+^ TEM (*p* = 0.038), and CD8^+^ naive (*p* = 0.004). When T cell subsets were analyzed by participants’ age, the same reduction in cell behavior was observed. However, no significant differences were observed among vaccination groups in the frequency of CD4^+^ (Table 2) and CD8^+^ T cell subsets (Table 3). Mean proportions and *p*-values are presented in Table 2 and Table 3, showing consistent levels across all groups. Figure 2 provides a representative analysis of memory T cells by flow cytometry. In addition to the number of specific T cell subsets, the activation status of T cells was evaluated in 25 random samples by quantifying CD69^+^ cells, an early marker of activation. The mean percentage of early-activated cells in the CD4^+^ and CD8^+^ subsets showed no significant difference between stimulated and unstimulated cultures (1.46% vs. 1.44% (*p* = 0.937) for CD4^+^ T cells and 2.52% vs. 2.47% (*p* = 0.937) for CD8^+^ T cells). The overall post hoc power analysis indicated an average statistical power of approximately 27%, corresponding to a small-to-moderate effect size (Cohen’s f ≈ 0.20).

**Figure 3:**
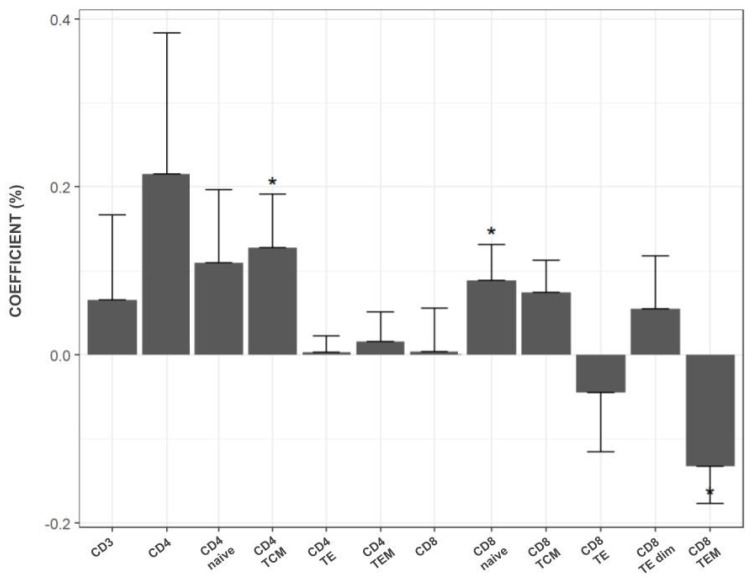
Estimated reduction in the percentage of memory T cell subsets in stimulated cultures every 30 days after the last received COVID-19 vaccine dose. The X-axis represents the CD4^+^ and CD8^+^ T cell subsets, while the Y-axis shows the coefficient, expressed as a percentage, calculated based on the mean percentage reduction for each T cell subset over time since the last COVID-19 vaccine dose. * indicates statistically significant differences (*p* < 0.05).

Regarding the time since the last vaccination dose, 94.4% of participants (51/54) had received their last COVID-19 vaccine at least two years prior to recruitment, while only 44.4% (24/54) had received it within the last 12 months. The average time since the last vaccine dose was 393 days (~13 months). A statistical model was applied to analyze the effect of the time since the last vaccine dose on T cell subsets in stimulated versus unstimulated cultures across the different vaccination groups. Figure 3 illustrates the estimated reduction in memory T cell subsets every 30 days. The highest rate of reduction was observed in the CD4^+^ subset, with statistically significant reductions in CD4^+^ TCM and CD8^+^ naive subsets (*p* = 0.013 and *p* = 0.020, respectively). Conversely, CD8^+^ TE and CD8^+^ TEM subsets showed an increase in quantity every 30 days in the presence of the SARS-CoV-2 stimulus, although statistical significance was observed only for the CD8^+^ TEM subset (*p* = 0.010).

### 3.3. rs2228059 Genotyping Findings

A total of 443 subjects from the Rio Grande do Sul population were included in the genotyping study: 62 participants recruited for the T cell memory evaluation and 381 subjects from HCPA Biobank. Genotyping analysis of the rs2228059 polymorphism was consistent with Hardy–Weinberg equilibrium (*p* = 0.738). The genotypic and allelic frequencies for both cohorts (recruited participants and HCPA biobank) are presented in Table 4. In our analysis, the minor allele frequency (MAF) of rs2228059 was 0.50 in the Biobank cohort and 0.484 in the study cohort, indicating very similar allele distributions between the two populations. All DNA samples showed adequate amplification and no ambiguous genotype calls were observed. No statistically significant differences were observed between stimulated and unstimulated conditions. An increase in CD8^+^ naive, CD8^+^ effector, and CD4^+^ naive subsets was observed in stimulated cells from participants in the ChAdOx1/ChAdOx1/BNT162b2 group with the TT genotype. However, the small sample size limits the ability to demonstrate statistically significant differences.

## 4. Discussion

Several studies have investigated the durability of cellular and humoral protection against SARS-CoV-2 by evaluating antibody titers in peripheral serum samples [13,28,29]. Measuring humoral antibody levels is relatively straightforward; however, memory B and T cells can persist even in the absence of detectable serum antibody levels [30]. Therefore, B and T cells, rather than circulating antibodies, play a crucial role in long-term immunity. In particular, pre-existing memory T cells specific to SARS-CoV-2 contribute to the direct immune response against the pathogen, support the maturation of the humoral immune response, and facilitate the establishment of specific memory B cells capable of responding rapidly to potential reinfections [31]. In this context, we analyzed cellular T-cell immune memory in individuals vaccinated with different regimens in Rio Grande do Sul, the southernmost state of Brazil.

In the scientific literature, immunological T-cell memory has been reported to persist for approximately 8 months following SARS-CoV-2 infection in 254 subjects [32,33,34]. The number of studies evaluating cellular memory is smaller compared to those focusing on humoral antibody titers. Only a few studies have combined the evaluation of antibody titers with cytokine measurements. In these studies, activation-induced marker (AIM) assays have emerged as the most frequently employed technique, often used in conjunction with enzyme-linked immunospot (ELISpot) assay for cytokine measurement [13,23,24,25]. The AIM assay involves incubating PBMCs with the antigen of interest under controlled cell culture conditions, thereby inducing the expression of antigen-specific markers indicative of a cellular response. Accurate detection of antigen-specific T cells depends critically on marker selection and stimulation duration [26]. Studies utilizing flow cytometry approaches to evaluate and quantify cellular memory have also been reported for other diseases. These methods offer the advantage of detecting specific immune responses independent of cytokine release [35].

To our knowledge, this study represents the most recent investigation of cellular responses in the post-pandemic period within a specific, diverse population and is the first to account for the particularities of the SARS-CoV-2 vaccination schedules administered. Evaluating specific regional populations is especially important in Brazil, a country of continental dimensions with extensive genetic diversity [21], as this diversity may contribute to potential regional variations in immune response formation to SARS-CoV-2. Our results did not reveal significant differences in cellular T-cell response profiles among different vaccination groups, suggesting that the preservation of cellular responses over time is independent of the vaccine schedule. Notably, we observed that the duration of cellular immune response declines progressively, with a reduction every 30 days after the last vaccine dose, regardless of the vaccine scheme received. This finding underscores the importance of timely booster vaccination campaigns and highlights the need to raise public awareness about the critical role of continuous vaccination in maintaining immune protection.

In this study, we applied a principle similar to the AIM assay, but memory T cells were directly evaluated by flow cytometry. A selection of markers recommended by the Euroflow guidelines [24] was used to assess lymphocyte ontogeny, and a peptide containing all antigens epitopes of SARS-CoV-2 was employed to stimulate T cells. Additionally, the cell culture medium was adapted to include autologous serum as a cell supplement, a strategy aimed at reducing nonspecific antigenicity. Specific SARS-CoV-2 cellular memory is predominantly stimulated by the immunological presentation of the spike protein epitope, which is the main target of neutralizing antibodies and the focus of therapeutic and vaccine design, despite the antigenicity of the entire viral structure [36]. Consequently, some vaccines, such as ChAdOx1-S and BNT162b2, are designed to mimic this process by targeting the spike protein [37]. In our study, we utilized a high-grade peptide pool, composed not only of spike proteins but also of 88 oligopeptides derived from the entire SARS-CoV-2 proteome [23]. However, this approach may result in understimulation of memory T cells at the applied concentration, potentially leading to an underestimation of antigen-specific CD8^+^ and CD4^+^ T-cell memory formed after vaccination. Although several studies use peptides at similar concentrations in AIM assays, there is considerable variability in the types of peptides employed for stimulation. Some studies use megapools containing structural proteins, others include both structural and non-structural proteins, and some focus exclusively on the spike protein for stimulation [38,39,40,41,42,43,44].

We observed that in the presence of SARS-CoV-2 peptides, there was a decrease in the overall number of CD3^+^ T cells, accompanied by a subtle increase in CD8^+^ TEM and TE subsets. This result suggests clonal expansion in response to the stimulus, as expected, when compared to the assay without stimulus across all vaccination groups. The marked decrease in CD4^+^ T-cell subsets may be attributed to exhaustion of T helper 2 (Th2) cells caused by overactivation, likely driven by clonal expansion of cells previously exposed to SARS-CoV-2. A similar phenomenon was observed in CD8^+^ T-cell subsets in another study [11]. CD4^+^ T memory cells, particularly circulating T follicular helper (cTfh) cells, play a critical role in the cellular immune response due to their ability to elicit antibody production. This behavior may help explain the statistically significant decrease in the CD4^+^ T-cell subset observed after incubation with viral antigens. Zhang and colleagues demonstrated, using a similar assay with longitudinal data and single-cell cytokine expression resolution, that CD4^+^ memory T cells responded to vaccine stimuli in a manner similar to their response to SARS-CoV-2 infection, even comparing different vaccine platforms [13]. Based on our data, we estimated the rate of decrease in T-cell subsets over time following the last booster vaccine dose. The majority of participants had received their final COVID-19 vaccination at least two years prior to recruitment, with fewer than half receiving their last dose within the 12 months preceding recruitment. Our analysis revealed that the CD4^+^ T-cell subset exhibited the highest variation among participants, with a decrease of 0.2% every 30 days after the booster, while CD8^+^ subset showed the lowest rate of decrease. These findings align with previous studies in the literature [11,13,34]. Despite this, the maintenance of long-term memory T cells may also depend on recurrent exposure to SARS-CoV-2 and/or periodic vaccination, which can expand the memory pool and contribute to more durable cellular memory [14]. Regarding T-cell activation as evaluated by CD69 expression, we analyzed 25 samples and did not observe differences before and after SARS-CoV-2 stimulation across all vaccination groups. The lack of observed activation could be attributed to the small sample size or the fact that patterns of CD69 upregulation are more pronounced in innate-like mucosa-associated invariant T (MAIT) cells compared to conventional CD4^+^ and CD8^+^ T cells, as shown in a study by Parrot and colleagues (2020) [45]. Notably, mucosa-associated cells were not evaluated in our study.

This study has certain limitations. The sample size did not provide sufficient statistical power to compare cellular responses among individuals who received the most common COVID-19 vaccine regimens administered in Rio Grande do Sul. While the overall post hoc analysis suggests limited sensitivity for detecting smaller effects, the preliminary nature of the study was sufficiently informative to highlight meaningful trends and significant differences in specific lymphocyte subpopulations. A larger sample size is needed to more effectively assess the potential influence of different vaccination regimens. However, the three vaccination groups analyzed had an equal age and sex distribution among participants, eliminating potential bias from these variables. Other studies evaluating cellular immunity against COVID-19 have also used small sample sizes [40,42,44], as they are primarily descriptive and do not focus on differences across vaccination regimens. Despite this, we were able to describe T-cell subsets before and after SARS-CoV-2 stimulation. The small sample size in our study, as well as in similar studies aiming to describe and quantify immune T-cell memory against SARS-CoV-2, reflects the complexity of the methodology required for this type of evaluation. Recruitment, cell isolation, culture, and treatment are labor-intensive processes that must be completed within a short timeframe, limiting the number of tests that can be conducted weekly. Although we observed statistically significant differences in the presence of stimulation, the small cohort size, particularly in the BNT162b2 group, may have contributed to the lack of observed differences in T-cell subsets among vaccination regimens. Finally, we evaluated only the cellular memory of circulating T cells. It is well-established that tissue-resident cells play a critical role in the lungs, particularly during acute infection, and that central memory T cells can potentially migrate to infected tissues [46].

Despite its limitations and preliminary nature of the results, this study provides valuable information, particularly when considering the significant impact of the pandemic in Brazil, including the state of Rio Grande do Sul. In Brazil, the slow pace of vaccination placed the country in a health crisis, with less than 25% of the population immunized during the first six months of the COVID-19 vaccination campaign [47]. The use of heterologous booster vaccinations with different platforms was a critical strategy during the pandemic, especially given the overwhelming demand and insufficient infrastructure to manage the crisis. However, this heterogeneity complicates the evaluation of immunological memory in the population. Studying the impact of heterologous SARS-CoV-2 vaccine boosters and their role in T-cell memory formation in the post-pandemic period remains essential for understanding long-term protection [48]. In addition to heterologous vaccination, other factors, such as genetic influences on the formation and persistence of memory cells, are important for understanding long-term immunity against SARS-CoV-2. Some studies suggest that IL-15, a cytokine involved in various immune processes, plays a significant role in the maintenance of memory T cells, particularly through its interaction with the IL15RA receptor subunit [49]. To contribute to this understanding, we genotyped the rs2228059 polymorphism to provide population data and support future research on how this polymorphism might influence long-term protection. This polymorphism may exhibit different frequencies across Brazilian states due to regional genetic diversity. In our study, we observed a higher frequency of the G allele (0.502), which is comparable to but slightly lower than the frequency reported in ABraOM (Online Archive of Brazilian Mutations) (0.523) [50]. Although the present study did not directly assess the functional impact of the rs2228059 polymorphism on IL-15 signaling, the similar distribution of the G and T alleles observed in our cohort suggests no substantial influence of this variant on the immune memory responses observed.

## 5. Conclusions

Since the onset of the pandemic, significant progress has been made in understanding the transmission and management of SARS-CoV-2 infections. In the current post-pandemic period, following the administration of multiple vaccine doses, investigating the persistence of cellular immune memory in the context of repeated COVID-19 vaccinations remains crucial. The findings presented here should be regarded as preliminary and exploratory. Nevertheless, we observed comparable characteristics in SARS-CoV-2-specific T-cell memory responses across different vaccine regimens. In addition, we demonstrated a decline in T-cell quantity over time following the last vaccine dose, which supports the relevance of booster doses not only against emerging variants but also to maintain long-term immune protection. Additionally, our study provides valuable data on the frequency of the rs2228059 polymorphism in our population. However, a larger cohort is needed for a more accurate assessment of T-cell immunity over time and the potential influence of this polymorphism on memory persistence.

## Figures and Tables

**Table 1:** Characteristics of the studied population, categorized by SARS-CoV-2 vaccination scheme.

	Group 1	Group 2	Group 3
Characteristic	CoronVac	ChAdOx1	BNT162b2
	Sinovac	Astrazeneca/Oxford	Pfizer/BioNTech
Individuals, *n*	23	24	7
Gender, *n* (%)			
Male	4 (17.4%)	7 (29.2%)	2 (28.6%)
Female	19 (82.6%)	17 (70.8%)	5 (71.4%)
Age, years (mean ± SD)	41.52 (±13)	39.54 (±15)	37.4 (±16)
COVID-19 previous infection, *n* (%)			
Yes	17 (73.9%)	20 (83.3%)	6 (85.7%)
No	6 (26.1%)	4 (16.7%)	1 (14.3%)
Months since last dose (median ± SD)	9 (±7)	13 (±5)	14 (±6)
Other COVID-like symptoms (last 6 months) *n* (%)			
Sore throat and cough	2 (8.7%)	1 (4.2%)	0
Flu-like	0	1 (4.2%)	0
Other viral disease	1 (4.3%)	0	0
Other bacterial disease	1 (4.3%)	2 (8.3%)	0
Other non-infectious comorbidities	2 (8.7%)	5 (20.8%)	1 (14.3%)
Asymptomatic	17 (74%)	15 (62.5%)	6 (85.7%)

*n* = number of participants; SD = standard deviation.

**Table 2:** Mean percentage of CD4^+^ T cell subsets in stimulated and unstimulated cultures, grouped by vaccination schemes. This table presents the comparative analysis of CD4^+^ T cell subsets (naive, TCM, TEM, etc.) in both stimulated and unstimulated conditions across the different vaccination groups. Statistical significance is indicated where applicable.

		CD4^+^ T Cell and Subsets											
		% CD3 Stimulated Cells	% CD3 Unstimulated Cells	% CD4 Stimulated Cells	% CD4 Unstimulated Cells	% CD4 Naive Stimulated Cells	% CD4 Naive Unstimulated Cells	% CD4 TCM Stimulated Cells	% CD4 TCM Unstimulated Cells	% CD4 TEM Stimulated Cells	% CD4 TEM Unstimulated Cells	% CD4 TEMRA Stimulated Cells	% CD4 TEMRA Unstimulated Cells
Group 1	Mean	*77.03*	*78.53*	*34.20*	*36.99*	*13.42*	*14.69*	*13.24*	*13.86*	*6.02*	*6.73*	*1.50*	*1.70*
	SD	*6.9*	*5.52*	*12.59*	*12.16*	*8.03*	*9.30*	*6.84*	*6.13*	*4.54*	*4.79*	*2.33*	*2.83*
Group 2	Mean	*78.00*	*80.17*	*39.98*	*42.74*	*17.33*	*18.13*	*15.53*	*16.54*	*6.41*	*6.69*	*1.45*	*1.40*
	SD	*8.13*	*8.94*	*13.03*	*11.24*	*10.06*	*9.92*	*5.24*	*4.91*	*3.62*	*3.45*	*1.67*	*2.01*
Group 3	Mean	*76.78*	*78.29*	*32.21*	*35.55*	*11.35*	*12.78*	*14.02*	*15.74*	*6.41*	*6.59*	*0.41*	*0.43*
	SD	*11.29*	*10.45*	*10.48*	*12.08*	*6.54*	*7.59*	*5.27*	*5.95*	*2.83*	*3.56*	*0.34*	*0.46*
Stimulated T cells (*p* value)		**0.005**		**0.007**		**0.045**		**0.021**		**0.038**		0.579	
Stimulated T cell vs. Vaccine (*p* value)		0.868		0.983		0.895		0.676		0.569		0.654	

TCM = central memory T cell; TEM = effector memory T cell; TEMRA = effector memory RA^+^ T cell. Bold values indicate statistically significant differences (*p* < 0.05)

**Table 3:** Mean percentage of CD8^+^ T cell subsets in stimulated and unstimulated cultures, grouped by vaccination schemes. This table compares the CD8^+^ T cell subsets (naive, TCM, TEM, etc.) under stimulated and unstimulated conditions across different vaccination groups. Statistically significant differences are highlighted where applicable.

		CD8^+^ T Cell and Subsets													
		% CD3 Stimulated Cells	% CD3 Unstimulated Cells	% CD8 Stimulated Cells	% CD8 Unstimulated Cells	% CD8 Naive Stimulated Cells	% CD8 Naive Unstimulated Cells	% CD8 TCM Stimulated Cells	% CD8 TCM Unstimulated Cells	% CD8 TEM Stimulated Cells	% CD8 TEM Unstimulated Cells	% CD8 TE dim Stimulated Cells	% CD8 TE dim Unstimulated Cells	% CD8 TEMRA Stimulated Cells	% CD8 TEMRA Unstimulated Cells
Group 1	Mean	*77.03*	*78.53*	*27.60*	*28.11*	*6.68*	*7.45*	*4.40*	*4.35*	*5.42*	*5.02*	*1.05*	*0.99*	*10.02*	*10.02*
	SD	*6.9*	*5.52*	*8.50*	*8.09*	*4.33*	*4.65*	*2.82*	*2.49*	*4.01*	*3.54*	*0.62*	*0.58*	*8.86*	*8.42*
Group 2	Mean	*78.00*	*80.17*	*26.12*	*27.25*	*7.65*	*8.53*	*4.91*	*5.39*	*3.68*	*3.56*	*2.16*	*1.38*	*7.70*	*8.37*
	SD	*8.13*	*8.94*	*6.52*	*7.49*	*3.53*	*4.42*	*2.71*	*3.26*	*2.02*	*2.00*	*4.23*	*0.63*	*5.59*	*5.90*
Group 3	Mean	*76.78*	*78.29*	*32.79*	*33.14*	*7.67*	*8.27*	*7.38*	*7.77*	*6.74*	*6.29*	*1.63*	*1.74*	*9.35*	*9.05*
	SD	*11.29*	*10.45*	*7.63*	*7.06*	*5.32*	*5.80*	*4.12*	*3.68*	*4.00*	*4.53*	*1.40*	*1.72*	*5.06*	*4.93*
Stimulated cells (*p* value)		**0.005**		**0.016**		**0.004**		0.315		0.327		0.346		0.541	
Stimulated cell vs. Vaccine (*p* value)		0.868		0.576		0.943		0.579		0.877		0.612		0.679	

TCM = central memory T cell; TEM = effector memory T cell; TEMRA = effector memory RA^+^ T cell; TE dim = effector T cell with reduced CD27 surface expression. Bold values indicate statistically significant differences (*p* < 0.05)

**Table 4:** Genotypic and allelic frequency results for rs2228059 polymorphism genotyping.

	Allele		Genotype		
	T	G	TT	GG	TG
Biobank cohort *n* = 381	381	381	93	95	193
Frequencies	0.500	0.500	0.244	0.249	0.506
%	50	50	24.4	24.9	50.7
Study participants cohort *n* = 62	60	64	14	16	32
Frequencies	0.484	0.516	0.225	0.258	0.516
%	48.4	51.6	22.6	25.8	51.6

## Data Availability

The raw data supporting the findings of this study are available from the corresponding author on reasonable request, including the sequencing raw data. All other data generated and analyzed during the realization of this study are included in this paper.

## Supplementary Materials

The following supporting information can be downloaded at: https://www.mdpi.com/article/10.3390/diagnostics16010089/s1, Supplementary Material Section S1: Supplementary Table S1. Reagents used for labeling in flow cytometry analyses; Supplementary Material Section S2: EuroFlow adapted protocol for surface marker staining.

## Abbreviations

The following abbreviations are used in this manuscript:

SARS-CoV-2	Severe Acute Respiratory Syndrome Coronavirus 2
COVID-19	Coronavirus Disease 2019
HCPA	Hospital de Clínicas de Porto Alegre
PBMC	Peripheral blood mononuclear cells
PHA-M	Phytohemagglutinin, M form
TCM	T cell central memory
TEM	T cell effector memory
TEMRA	T cells effector memory expressing RA^+^
TE	T cell effector memory
STREGA	STrengthening the REporting of Genetic Association Studies
AIM	Activation-induced marker
ELISpot	enzyme-linked immunospot
Th2	T helper 2
cTfh	Circulating T follicular helper
MAIT	Mucosa-associated invariant T
ABraOM	Online Archive of Brazilian Mutations
