# Supplementary material for: Cellular Immunological Memory T Cells and IL15RA Gene Polymorphism in COVID-19 Vaccinated Individuals from Southern Brazil
Source: Diagnostics (Basel). 2025 Dec 26;16(1):89. doi: 10.3390/diagnostics16010089 (PMC12785960; doi:10.3390/diagnostics16010089)
Supplement: Supplementary file 1 [file diagnostics-16-00089-s001.zip › Supplementary material (1).pdf]

Antibody		Fluorochrome	Clone	Source	Identifier	Panel	Volume
CD45	BD Multitest	PerCP	2D1	BD Biosciences	340499	1 and 2	5 µL
CD3	BD Multitest	FITC	SK7	BD Biosciences	340499	1 and 2	
CD4	BD Multitest	APC	SK3	BD Biosciences	340499	1 and 2	
CD8	BD Multitest	PE	SK1	BD Biosciences	340499	1 and 2	
CD27	BD Pharmingen APC-H7	APC-H7	MT271	BD Biosciences	560222	1	2.5 µL
CD45RA	BD CD45RA PE-Cy7	PE-Cy7	L48	BD Biosciences	649457	1	2.5 µL
CD69	APC/Cyanine7 anti-human CD69 Antibody	APC-Cy7	FN50	BioLegend	310913	2	2.5 µL

Supplementary Table S1. Reagents used for labeling in flow cytometry analyses.

SUPPLEMENTARY MATERIAL 2:

EuroFlow adapted protocol for surface marker staining

The surface marker staining protocol used in this study was adapted from the flow cytometry panel described in the study "*The EuroFlow PID Orientation Tube for Flow Cytometric Diagnostic Screening of Primary Immunodeficiencies of the Lymphoid System*", as outlined below:

After incubation for 20–24 hours at 37°C with 5% CO_2 in a humidified incubator, cells were harvested using the trypsinization protocol and collected into 1.5 mL microtubes. For each culture plate well, five cytometry tubes were prepared and labeled, as illustrated in Supplementary Figure 1. Additionally, an unstained tube was prepared for each study participant to adjust the cytometer voltage.

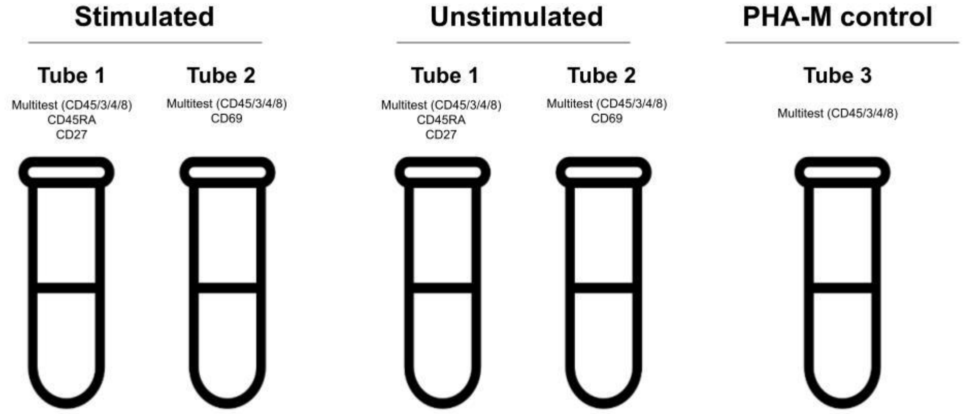

Supplementary figure S1. Representation of cytometry tubes with specific antibodies for each panel.

The cell-containing microtubes were centrifuged at 3,000 RPM for 5 minutes, and the supernatant was discarded. The cell pellet was resuspended in 100 µL of PBS—50 µL was transferred to tube 1, and 50 µL to tube 2. In tube 1, 5 µL of the multitest (CD 45/3/8/4) and 2.5 µL of CD27 and CD45RA were added. In tube 2, 5 µL of the multitest (CD 45/3/8/4) and 2.5 µL of CD27 and CD45RA were added, followed by gentle vortexing. In tube 3, only 5 µL of the multitest (CD 45/3/8/4) was added, along with 50 µL of cells from the PHA-M well plate culture. The remaining 50 µL was used for the unstained control tube.

Tubes were incubated for 30 minutes at room temperature, protected from light. Following this, 500 µL of FACSLysing (already diluted 1:10) was added, gently vortexed, and incubated for 10 minutes at room temperature, protected from light.

Subsequently, the tubes were centrifuged for 5 minutes at 540 g, and the supernatant was carefully discarded, leaving approximately 50 µL at the bottom. It is essential to handle this step carefully to avoid losing cells.

Afterwards, 2 mL of PBS was added, gently vortexed, and centrifuged for 5 min at 540 g. The supernatant was carefully discarded, leaving approximately 50 µL at the bottom. Tubes were gently vortexed, and 500 uL of FACSFlow was added. A minimum of 10,000 events must be acquired to ensure a sufficient number of events in lymphocyte gate.
